# Research on prevention and control technology of classified rockburst in TBM construction of deeply buried tunnels

**DOI:** 10.1038/s41598-024-51228-y

**Published:** 2024-01-03

**Authors:** Yalei Yang, Lijie Du, Qingwei Li, Cheng Gong, Yin Song, Minyuan Wang

**Affiliations:** 1 School of Mechanical Engineering, Shijiazhuang Tiedao University, Shijiazhuang, 050043 China; 2 Collaborative Innovation Center for Performance and Safety of Large-Scale Infrastructure, Shijiazhuang Tiedao University, Shijiazhuang, 050043 China; 3 PowerChina Chengdu Survey and Design Research Institute Co. Ltd., Chengdu, 610072 China; 4 School of Safety Engineering and Emergency Management, Shijiazhuang Tiedao University, Shijiazhuang, 050043 China

**Keywords:** Natural hazards, Engineering

## Abstract

Rock blasting and other geological disasters occur frequently in the TBM construction of deeply buried tunnels and seriously threaten construction safety and progress. Therefore, it is extremely important to conduct scientific research for effective prevention and control of rockbursts in construction. Based on a large number of field rockburst data, this study analyses the influence of rockburst on construction safety and efficiency by using statistical theory and summarizes the temporal and spatial characteristics of rockburst time, location and influence range. Using these results, combined with the characteristics of the TBM structure and construction method, classification prevention and control objectives, theoretical criteria and prevention and control technology of rock bursts are proposed. A theoretical system of classified prevention and control of rockburst is constructed, which is cooperatively controlled by microseismic monitoring, TBM equipment, TBM excavation and support measures. The system is verified to provide practical protection, demonstrating that this report provides an important reference for the prevention and control of rock bursts in ultradeep tunnels.

## Introduction

With the full-face rock tunnel boring machine (TBM) being widely used in various infrastructure fields, it is also facing new challenges in the construction of deeply buried tunnels^1,2^. The geological conditions of deeply buried tunnels are complex and have high ground stress^3^. Serious rockburst geological disasters easily occur, posing a serious threat to the construction progress of TBMs and the safety of personnel and equipment^4,5^. For example, a large number of strong rockbursts or even extremely strong rockbursts occurred during the excavation of the Jinping II Hydropower Station. This caused two TBMs in the diversion tunnel to stop construction actively, and one TBM in the drainage tunnel was destroyed and buried, causing casualties and enormous economic losses^6^. The Yinhanjiwei project (YHJW) also encountered a strong rockburst under construction. In early construction, the existing support was destroyed many times, and the TBM equipment was severely damaged by rockburst impact. This phenomenon greatly affected construction safety and progress^7^. Two TBM tunnels of Pakistan's N–J hydropower project suffered frequent rockbursts, causing casualties and serious equipment damage^8^. Rockburst also caused casualties during the TBM construction of the Kobbelv HPS water conveyance tunnel in Norway^9^. Therefore, it is of great significance to study the prevention and control technology of rock bursts in deeply buried tunnels to ensure the safety and economic construction of tunnels.

With the recent development of underground engineering construction in the direction of long, large and deep construction, the prevention and control of rockburst and rockburst monitoring has attracted increasing attention from scholars. Some reports have studied and proposed prevention and control measures to address rockbursts. In the aspect of rockburst prevention and control, He et al.^10^ proposed the energy absorption compensation technology of rock bursts. That is, a supporting material with Gao Heng resistance, large deformation, anti-impact and anti-explosion properties, and super energy absorption was used to support the surrounding rock of the tunnel in time with high pretightening. In view of rock burst control technology, Li et al.^11^ proposed some prevention and control measures, such as spraying cold water, evenly injecting water into drilling holes, short-footage excavation and plum-blossom anchor net support. According to the problem of rockburst, Tang et al.^12^ proposed prevention and control measures such as stress release, dense bolt support and flexible energy-absorbing protective nets. Based on the rockburst initiation process, Feng et al.^13^ proposed that rockburst prevention and control measures should be determined according to the rockburst grade of microseismic monitoring and early warning. Guo et al.^14^ analysed the failure characteristics of a medium rockburst zone under different surrounding rock conditions of a deeply buried TBM tunnel. The prevention and control support scheme of the medium rockburst area is established to distinguish the surrounding rock conditions. Ouyang et al.^15^ combined active and passive prevention and control measures for rock bursts during the construction of deeply buried tunnels. Active and passive combined prevention and control measures are proposed. The core of active measures is prestress release. In passive measures, a shotcrete anchor net and steel arch frame are adopted to strengthen the surrounding rock, and the fast and flexible support type is emphasized. In the aspect of rockburst monitoring, Ma et al.^16^ proposed a rockburst criterion based on the N–J hydropower project in Pakistan. The rockburst can be judged according to the ratio of the strength of the rock mass to the horizontal stress perpendicular to the axis of the tunnel. In the aspect of rockburst microseismic monitoring, Zhao et al.^17^ relied on the monitoring data of rockburst microseisms and rockburst cases of the Jinping II hydropower station project to analyse the temporal and spatial law of rockburst from the perspective of microseismic monitoring results. This shows that microseismic events have strong temporal and spatial correlations with rockbursts. Chen et al.^18^ analysed the law of microseismic activity in the process of TBM excavation and found that the faster the TBM excavation speed is, the stronger the microseismic activity. The high incidence period of rockburst is 4–6 h when the tunneling starts again after TBM maintenance. Wang^19^ calculated statistics on the monitoring results of rockburst microseisms and rockburst conditions in the upstream and downstream of tunnels No. 3 and No. 4 of the Qinling Tunnel from the YHJW project and found that the prediction accuracy rates were 95.89%, 90.00% and 88.46%, respectively. Ma et al.^20^ analysed the rock bursts in the Jinping II hydropower station project and found that the accuracy of rock burst prediction in this project can reach 80.6%. On the basis of microseismic monitoring and big data analysis of actual rockburst, Yang et al.^21^ found that the higher the rockburst grade is, the higher the accuracy of microseismic monitoring, and the accuracy of strong rockburst risk grade prediction can be close to 80%. Generally, this prior research on rockburst monitoring and rockburst prevention and control has been studied and discussed from different perspectives and has achieved fruitful research results. However, research on rock burst prevention and control technology for the TBM construction method is less comprehensive. Specifically, there is a lack of systematic technical system guidance for the prevention and control of different rockburst grades. Moreover, the rationality and effectiveness of relevant prevention and control technical schemes lack practical engineering application verification.

Therefore, this paper presents a statistical analysis based on the TBM construction site data of the YHJW Project and Xinjiang Aibihu Project (ABH). Combined with the structural features and construction methods of TBM equipment, the classification prevention and control objectives, prevention criteria and prevention and control technologies of TBM rockburst are systematically proposed on the basis of summarizing and analysing the characteristics of TBM rockburst. The results are verified in the ABH project and the project of diverting water from Han to Wei, which provides a reference for the prevention and control of rock bursts in TBM construction of deeply buried tunnels.

## Project overview

### Project overview

**Figure 1:**
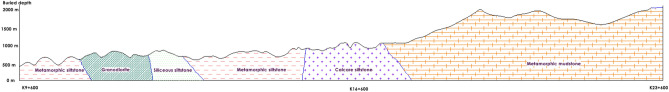
ABH project.

The total length of the main tunnel of the Xinjiang ABH project is approximately 41 km, of which 9 km is excavated by drilling and blasting, and 32 km is excavated by two open TBMs. The tunnel is located in the strong uplift area of the northern Tianshan Mountains, with developed folds and faults, strong seismic activity and high ground stress. Hard and brittle rocks such as siltstone, metamorphic mudstone and granodiorite are distributed along the line of the tunnel, and the compressive strength of the rocks is 55.6–148.7 MPa, which represents geological conditions that promote rockburst. The longitudinal section of the ABH engineering geology is shown in Fig. 1.

**Figure 2:**
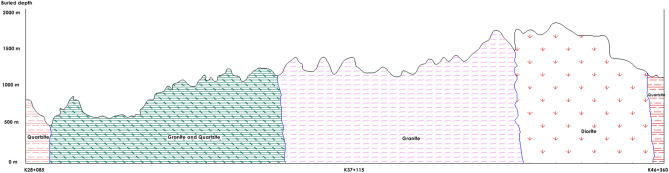
YHJW project.

The YHJW project is located in the Qinling Mountains in south-central Shaanxi Province, China. The total length of the project is 98.3 km, which is excavated by the drilling and blasting method and two 8.0 m diameter open-type TBMs. The elevation of the tunnel ranges from 1050 to 2420 m, the maximum burial depth is approximately 2012 m, and the ground stress is high. Hard rocks such as quartzite, granite and diorite are mainly distributed along the line of the tunnel. The compressive strength of rock is 107–317 MPa, and the average value is approximately 160–170 MPa, which represents favourable conditions for strong rockburst. The longitudinal section of the YHJW engineering geology is shown in Fig. 2.

### Rockburst situation

The excavation pile number of section III of the ABH project studied in this paper is K9 + 600-K23 + 600. Up to August 2019, there were 151 rockbursts during the TBM excavation of this project, including 101 slight rockbursts, 18 slight to moderate rockbursts and 32 moderate rockbursts. There was no occurrence of strong or extremely strong rockburst. Thus, the impact of rockburst on the construction is minimal.

The total length of the Lingnan section of the YHJW project studied in this paper is 18.28 km, and the pile numbers are K28 + 085 − K46 + 360. Among these piles, the number K28 + 490 − K37 + 011.5 represents the first tunneling section of the TBM, and the number K39 + 511 − K46 + 360 represents the second tunneling section of the TBM. Up to November 2019, there were 795 rockbursts during TBM excavation, including 302 slight rockbursts, 84 slight to moderate rockbursts, 158 moderate rockbursts, 80 moderate to strong rockbursts and 171 strong rockbursts. It has a great impact on TBM construction.

## Impact analysis of rockburst on TBM construction

### Impact of rockburst on TBM construction safety

**Figure 3:**
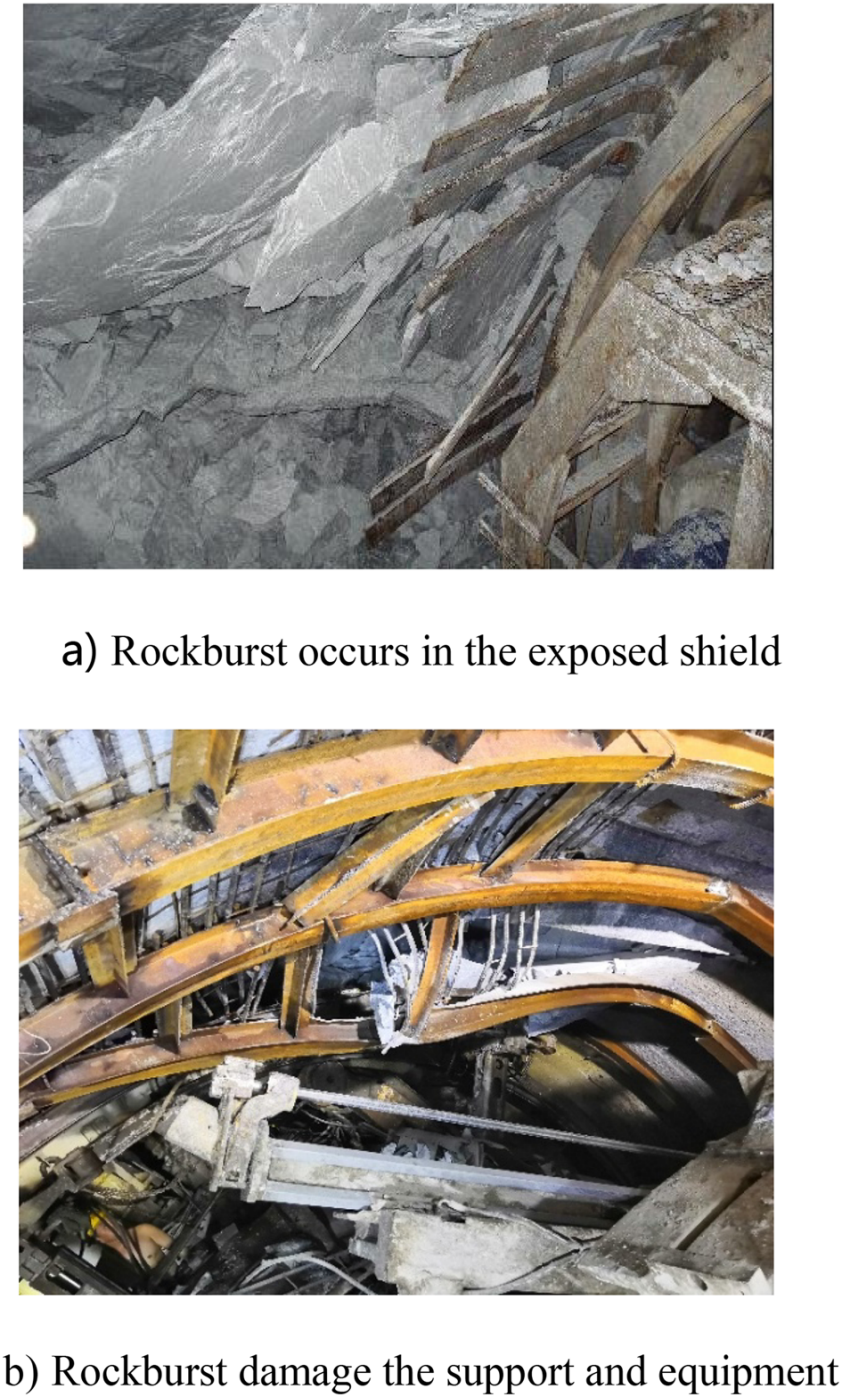
Impact of rockburst on TBM construction.

Once a rockburst occurs during TBM construction, it will slow the construction speed and threaten construction safety. Moreover, the impact of different grades of rockburst on TBM construction is generally different. The impact of rockburst on construction is shown in Fig. 3.

According to the analysis and summary of the site situation of rock bursts in the two supporting projects, the influence law of rock bursts on construction safety is as follows.

1. The energy of slight and slight-moderate rockbursts is low. After rockburst, local rocks in the tunnel burst, resulting in less rock slag. It will not destroy the initial supporting system and has little influence on the construction personnel and equipment.
2. The energy of moderate and moderate-strong rockbursts is higher. After the rockburst, more rock slag is produced. However, generally, it will not destroy the initial support system. Due to the buffer of the supporting system, the equipment is not damaged by falling rocks. We should guard against personal injuries caused by rockbursts and falling rocks.
3. Strong rockbursts and extremely strong rockbursts produce strong energy. After the rockburst, there are many rocks with strong impacts. It poses a great threat to personnel and equipment safety. If this type of rockburst occurs behind the shield, most of the initial support system will be destroyed, the equipment may be damaged, and even the equipment and engineering will be destroyed in severe cases. If strong rockburst occurs in the area from the shield to the tunnel face, then the shield, main drive and cutter head will certainly incur at least some impact or damage. Simultaneously, the abnormal damage of the disc cutter and bucket on the cutter head will greatly increase.

### Impact of rockburst on TBM construction efficiency

The construction speed of the TBM, i.e., the feed rate is mainly affected by both the pure digging speed and the pure digging time of the TBM. It is mainly affected by the TBM pure advance speed and pure tunneling time. After classifying and summarizing the construction parameters of the rock burst section, we can obtain the influence law of rock burst on the TBM construction speed by comparison with the construction parameters of the nonrock burst section. After the statistical analysis of the types of surrounding rock with rockburst risk in the two supporting projects, it is found that the Class IV surrounding rock with rockburst risk is only approximately 150 m, and only 20 rockbursts have occurred. Due to the lack of rockburst data in Class IV surrounding rock, this paper only discusses the influence of rockburst on the TBM construction speed under the conditions of Class I, Class II and Class III surrounding rock.

#### Influence of rockburst on the TBM pure advance speed

**Figure 4:**
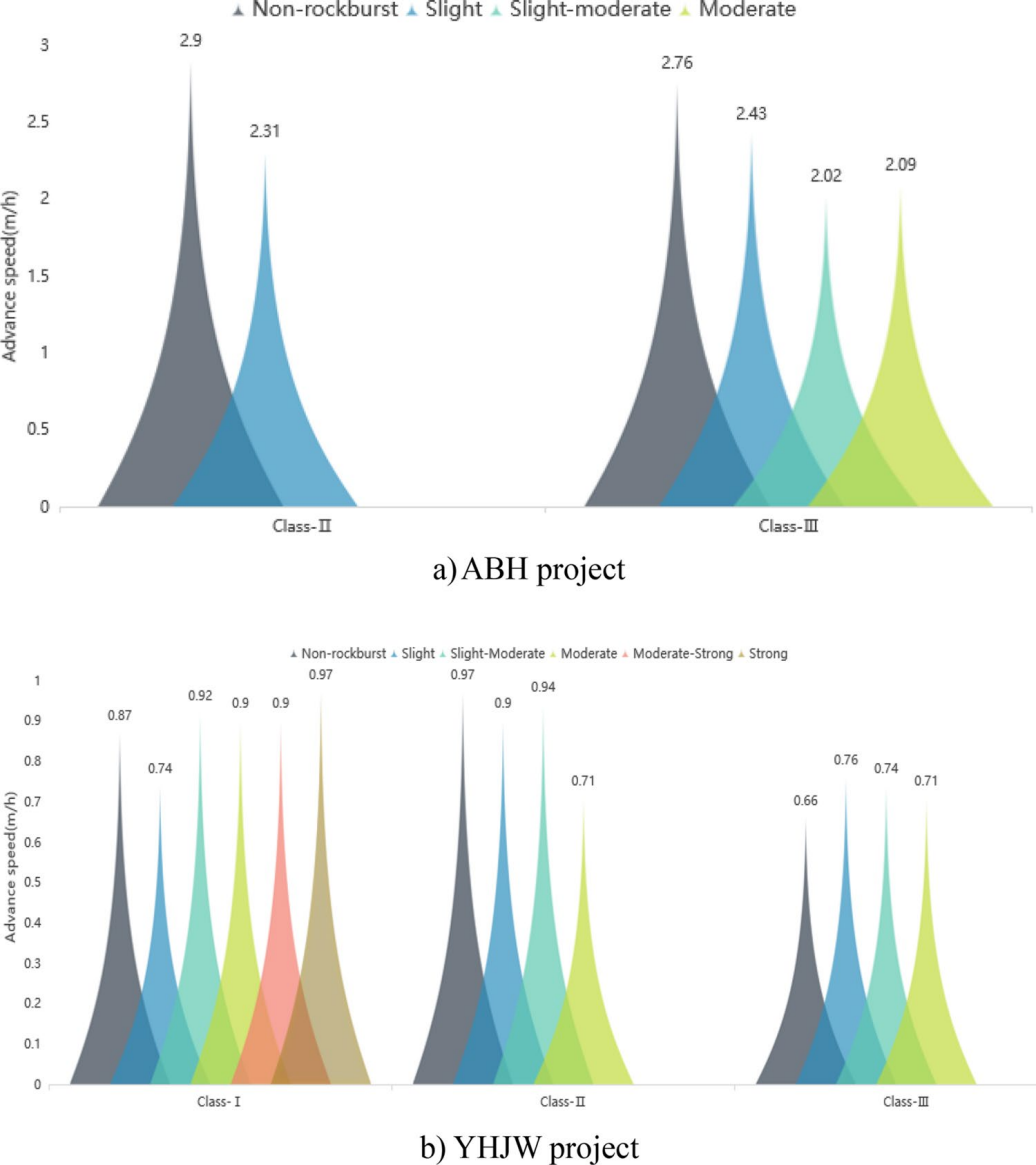
Comparison of advance speed under different surrounding rock classification and different rock burst grades.

The speed of the TBM in the rockburst section and nonrockburst construction tunnel section in the two supporting projects is counted. The influence of rockburst on the TBM advance speed is analysed. The results are shown in Fig. 4.

Figure 4a shows that the advance speed of the TBM is clearly reduced due to the occurrence of rockburst in the ABH project. In a certain range, the influence of rockburst on the TBM advance speed is gradually strengthened with the improvement of the rockburst grade. The main reason is that the compressive strength of rocks is low in the ABH project, which is suitable for TBM excavation. If there is no rockburst, then the pure advance speed of the TBM is faster. After a rock burst occurs, the advance speed will be clearly reduced. This is mainly due to the active control of the TBM advance speed by the rock burst tunnel section. However, Fig. 4b shows that the influence of rock bursts on the TBM advance speed is not clear in the YHJW project. In some cases, the advance speed of the rock burst section is even slightly higher than that of the nonrock burst section. Only from the statistical results of this project can it be found that the correlation between the advance speed and rockburst grade is weak. This occurs because the rock compressive strength is extremely high in the YHJW project. Even without rockburst, the pure advance speed of the TBM is very low. After the rock burst occurs in this extremely hard rock, the pure advance speed will not increase or decrease clearly, and it does not need to be actively controlled. Therefore, the easier it is for the surrounding rock to penetrate, the greater the proportion of rock burst will affect the construction speed of the TBM. Moreover, the main factor affecting the construction speed of the TBM is not the pure advance speed but the pure advance time.

#### Influence of rockburst on the TBM pure advance time

**Figure 5:**
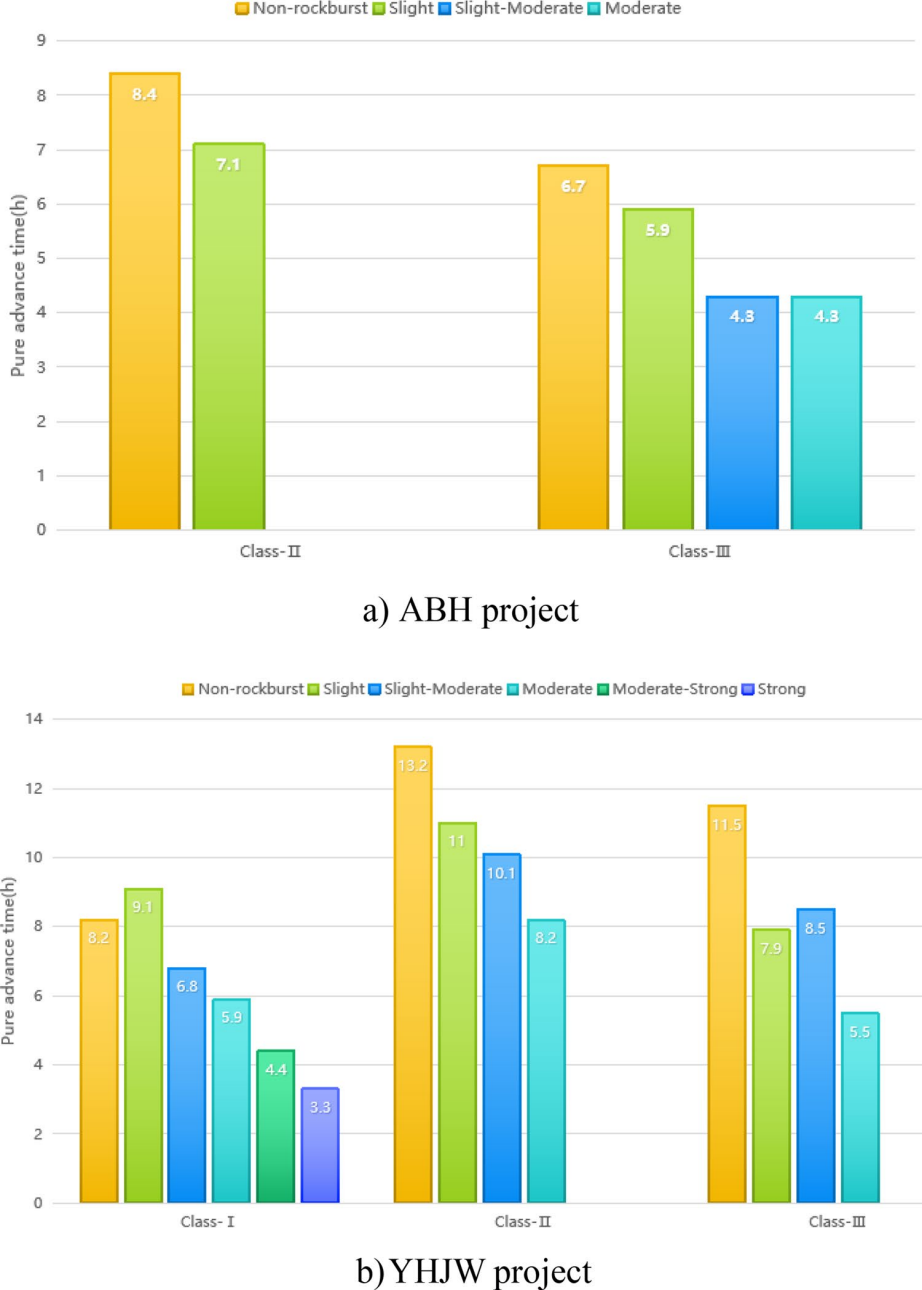
Comparison of pure advance time under different surrounding rock classification and different rock burst grades.

Rockburst will increase the workload of slag cleaning, support and equipment repair. This workload includes a pure advance time, which further affects the penetration speed of TBM construction. Generally, under the same type of surrounding rock, the higher the rockburst grade is, the greater the delay of the pure advance time. The pure tunneling time of the TBM in the rock burst section and nonrock burst construction section of the two supporting projects is counted. The influence of rockburst on the TBM pure tunneling time is analysed, as shown in Fig. 5.

#### Influence of rockburst on the TBM construction speed

The daily footage of the TBM is counted in the rock burst section and the tunnel section without rock bursts in the two supporting projects. This paper analyses the influence of rock bursts at all levels on the construction speed of TBMs under different surrounding rock classifications. Taking the construction speed of the nonrockburst section as a reference, the construction speed is expressed as 100%. Then, under the influence of different grades of rockburst, the percentage of construction speed of the rockburst section is the nonrockburst section, as shown in Table 1.

**Table 1:** Comparison of construction speed between rock burst section and nonrock burst section.

Project	Class	Nonrockburst (%)	Slight (%)	Slight-moderate	Moderate	Moderate-strong	Strong
ABH	II	100	67.8	–	–	–	–
	III	100	77.2	49.5%	48.4%	–	–
YHJW	I	100	95.8	87.3%	74.6%	54.9%	45.1%
	II	100	76.7	73.6%	45.0%	–	–
	III	100	78.9	82.9%	51.3%	–	–

The above construction speed considers the influence of rock burst prevention and control support measures. From Based on statistical analysis in Table 1, certain conclusions are made as follows. (1) In the same project with the same rockburst grade, the influence of rockburst on the construction speed of Class II surrounding rock is more clear than that of Class III. Under the condition of similar surrounding rock of different projects, the influence of rock bursts of different grades on the construction speed of the ABH project is more clear than that of the YHJW project. The reason is that the rock strength of the ABH project is low, and it is easy to penetrate. If there is no rockburst, the supporting capacity of the Class II surrounding rock is very small. A very high advance speed can thus be obtained. If there is the possibility of rockburst, then it is necessary to take support measures similar to broken surrounding rock. This measure will increase the impact on the proportion of pure advance time. Therefore, rockburst has a great influence on the construction speed. (2) The active control of daily footage in Class I surrounding rock is not considered in the YHJW project. When moderate rockburst occurs in two projects, the construction speed is only approximately 1/2–3/4 of that without rockburst. This shows that rockbursts have a great influence on construction speed. (3) Within the scope of the tunnel studied in this paper, strong rockburst occurred only under the condition of Class I surrounding rock in the YHJW project. The construction speed of the strong rockburst tunnel section is less than 1/2 of that of the nonrockburst tunnel section. This shows that rockbursts have a great influence. If the same strong rockburst occurs in the ABH project, because the rock strength is lower than that of the YHJW project, signifying that it is easy to penetrate and that the pure tunneling speed is high. Moreover, there is a large gap between the pure tunneling time with strong rockburst and that without strong rockburst. Therefore, if a strong rock burst occurs in an ABH project, the influence degree on the penetration speed of TBM construction is far more than half. In fact, especially for the prevention and control of strong rockbursts, preventive measures are generally taken in advance. Therefore, even if the Class I surrounding rock of the YHJW project is a normal tunnel section without rockburst, the measures of actively controlling the daily footage are sometimes taken. Therefore, the impact of rockburst on the construction speed may be greater than the statistical analysis in Table 1. The support of Class I surrounding rock is very simple when the nonrockburst section of the YHJW project is constructed without considering the control of footage. Theoretically, the pure tunneling time is approximately 15 h, and the daily footage can reach 12.9 m. Under the same lithology and compressive strength, the actual daily footage of the strong rock burst tunnel section is 3.2 m. Therefore, the construction speed of the strong rock burst section may be only approximately 1/4 of that of the nonrockburst section.

## Temporal and spatial characteristics of rockburst

The characteristics of rockburst in TBM construction should be accurately acquired for rockburst prevention and control. This study focuses on analysis of the “space–time effect” of rockburst. That is, the characteristic law of rockburst is analysed from three aspects: the occurrence time (tunneling lag time), the occurrence position (the distance to the heading face) and the influence range. Due to the low rockburst grade of the ABH project, there is no rockburst with a medium or above grade. There are few rockburst datasets, so the accuracy of the conclusion may be biased. Such a result would render the corresponding research less useful. Therefore, the following discussion only summarizes the temporal and spatial characteristics of rockburst in the YHJW project.

For statistical convenience, T is used to represent the lag time of rockburst (that is, the time from the excavation of a certain pile number to the occurrence of rockburst). According to the construction time of the project, T is grouped with 12 h, 24 h, 48 h and 72 h as nodes. S is used to represent the distance from the position of the rockburst to the working face. According to the structural characteristics of the TBM, 5 m, 15 m and 60 m are used as nodes for grouping. D is used to represent the influence range of the rockburst (including the length of the rockburst section and the circumferential position of the rockburst). Among these values, the length of the rockburst section is the distance from the starting pile number to the ending pile number of the rockburst location. The location of rockburst refers to the location of rockburst on the circumference of the tunnel section. The upper 120 range is divided into the top arch, the lower 120 range is the bottom arch, and the left and right sides are the sidewalls.

### Statistical analysis of rock burst characteristics

**Figure 6:**
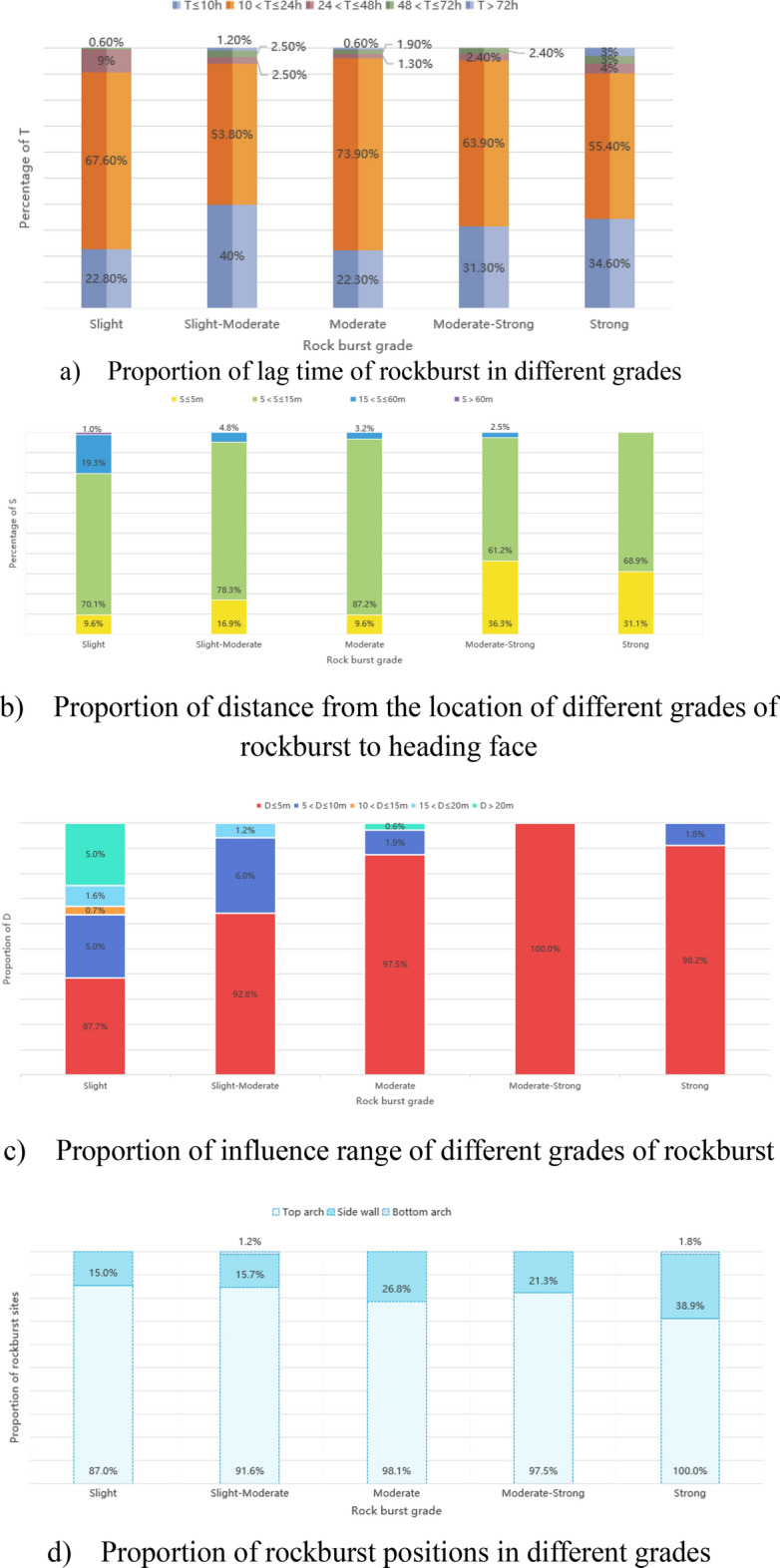
Temporal and spatial characteristics of rockburst.

Because some rockburst datasets are incomplete, 788 groups of relatively complete rockburst data are selected for analysis in the YHJW project, including 301 minor rockbursts, 83 minor-moderate rockbursts, 157 moderate rockbursts, 80 moderate-strong rockbursts and 167 strong rockbursts. The temporal and spatial characteristics of rockburst are obtained by statistical analysis of T, D and S in these data. The relatively complete rockburst data recorded in the YHJW project are classified according to the rockburst grade. T, D and S of rockburst at all levels are statistically analysed, and the statistical results are shown in Fig. 6.

The statistical analysis results in Fig. 6 show that the rockburst that occurred in the YHJW project has the following characteristics. (1) More than 90% of rockbursts occur within 24 h, the frequency of rockbursts is the highest within 10–24 h after excavation, and approximately 9% of strong rockbursts occur within 24–48 h. Therefore, the key prevention and control time of rockburst should be within 24 h after excavation. (2) The distance from the rockburst position to the tunnel face is mostly 5–15 m. With increasing rockburst grade, the distance from the rockburst to the heading face decreases. The proportion of strong rockburst before the shield is 31.1%. According to the characteristics of an open TBM, the main girder is usually located within the range of 5–15 m from the tunnel face. The personnel and equipment here are relatively concentrated, so the prevention and control of rockburst must be performed well. (3) The length of a single rock burst section is mostly within 5 m. One hundred percent of moderate to strong rockbursts and 98.2% of strong rockbursts are in the range of 5 m. Rockbursts mostly occur within 120 of the top arch, and 98.1%, 97.5% and 100% of moderate, moderate-strong and strong rockbursts occur in the top arch, respectively. There are 15.9%—38.9% rockbursts on both sidewalls. There are cases where the top arch and the sidewall rock burst simultaneously. Rock bursts at the bottom arch account for very little. Therefore, the key prevention and control positions can be determined according to the influence range of rockbursts at all levels. Simultaneously, the support of the rock burst section should be mainly within 120 of the top arch.

### Characteristics of strong rockbursts controlling TBM daily footage

**Figure 7:**
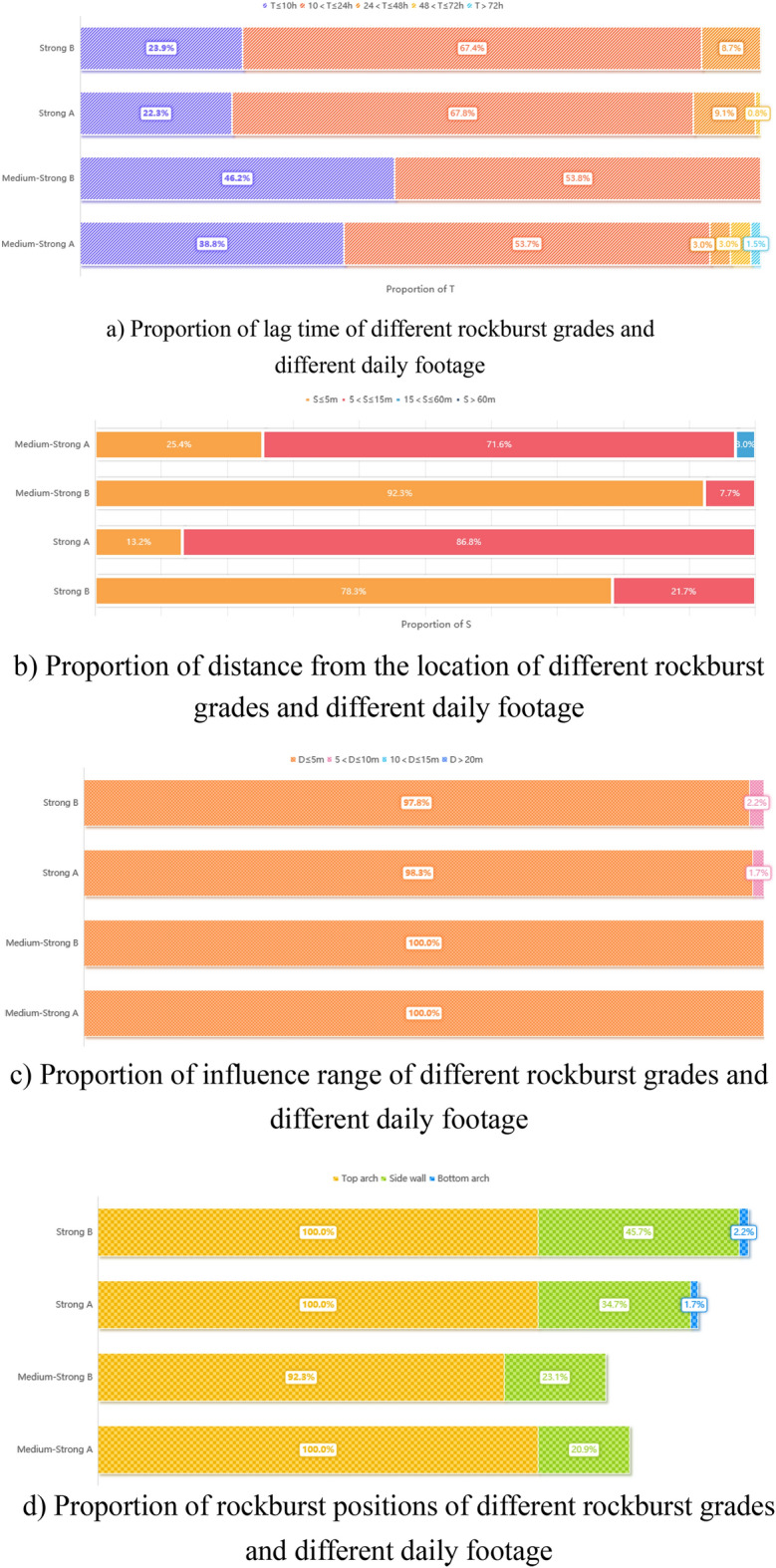
Characteristic of strong rockburst controlling TBM daily footage.

The influence of strong rockburst is reduced by actively controlling the daily footage of TBM excavation in the YHJW project. To clarify its prevention and control effect, the moderate ~ strong and strong rockbursts in this project are verified in groups with a daily footage of 3 m as the node. In the selected data of this project, there are 80 moderate to strong rockbursts and 13 instances of daily footage below 3 m. There were 167 strong rockbursts, and 46 instances of daily footage below 3 m. Data with daily footage above 3 m can be represented by medium-strong A and strong rockburst A, and data with daily footage below 3 m can be represented by medium-strong B and strong rockburst B. The statistical results are shown in Fig. 7.

Figure 7 shows that after the daily footage is reduced, more than 90% of rockbursts still occur within 24 h, and there is no clear change in T and D, but S is significantly reduced. The proportions of moderate-strong and strong rockbursts occurring within 5 m (i.e., from shield to tunnel face) increased clearly, reaching 92.3% and 78.3%, respectively. This is of great significance to the prevention and control of rock bursts in TBM construction. For an open TBM, the distance from the tunnel face to the tail of the shield is generally approximately 6 m. The shield supports the cave wall. Thus, by controlling the daily footage to approximately 3 m in the YHJW project, most of the strong rockbursts can occur within the shield and tunnel face. To avoid the damage of support or equipment caused by strong rock bursts and reduce the influence caused by rock bursts. For other projects, the daily footage can also be actively controlled according to the specific lithology, rock burst strength and characteristics; for example, the daily footage of the strong rock burst section can be controlled in the range of 3–55 m, and the specific daily footage can be determined according to the actual field test of the project.

## Prevention and control of rockburst

Concepts and technologies for rock burst prevention and control in TBM construction are proposed based on the present understanding of the characteristics and laws governing rock burst, combined with the structural characteristics and construction methods of TBM.

### Target of rock burst prevention and control in TBM construction

As a natural geological phenomenon under certain conditions, rockburst has a large impact energy when it is strong, and its occurrence is sudden and uncertain. Although some prediction theories and monitoring techniques exist to support this goal, it nonetheless remains difficult to accurately determine when, where and how much energy rockburst will occur in advance. Even if the occurrence of rockbursts is predicted accurately, other reliable prevention and control technologies need to be implemented to ensure the safe crossing of TBMs. Therefore, the prevention and control objectives of rockburst must be objective and realistic, and the benefits of safety and speed must be fully considered according to the grade of rockburst. Toward providing solutions for various levels of rockburst based on the influence degree of the delay of advance speed, the workload of support and later recovery, and the harm of equipment and personnel, the following prevention and control objectives of rockburst classification are proposed.

1. Prevention and control targets of slight rockburst. After the timely and reliable implementation of prevention and control technical measures, the influence of similar class-III surrounding rock support on TBM excavation delay was determined. Rock bursts and the implementation of prevention and control measures have little impact on TBM footage speed. Rock burst and collapse in the cave wall can be controlled without damaging the supporting structure. There is no extra slag cleaning workload to avoid the damage of rock bursts to equipment and personnel.
2. Prevention and control targets of medium rockburst. After timely and reliable implementation of prevention and control technical measures, the influence of similar class-IV surrounding rock support on TBM excavation delay. Rock bursts and the implementation of prevention and control measures have a great influence on the penetration speed of TBMs. The cave wall burst and cave-in and rock ejection were basically controlled. The implemented supporting structure will not be damaged. There is basically no extra slag cleaning workload. The damage of rockbursts to equipment and personnel should be avoided.
3. Prevention and control targets of strong rockburst. After timely and reliable implementation of prevention and control measures, the influence of similar class-V surrounding rock support on TBM excavation delay. Rock bursts and the implementation of prevention and control measures have a great influence on TBM footage speed. The deep blasting, impact ejection and falling rock of the cave wall are controlled to a certain extent. The supporting structures in individual positions are damaged, and the workload of restoring support is not great. Rock bursts should be avoided from causing great damage to equipment. Avoid personal injury.
4. Prevention and control targets of extremely strong rockburst. After timely and reliable implementation of prevention and control measures, the influence of similar V-level extremely broken surrounding rock support on TBM excavation delay. Rock bursts and the implementation of prevention and control measures have greatly reduced the penetration speed of TBM construction. The rock mass collapsed on a large scale, and the supporting structures in individual positions were damaged, so the workload of slag cleaning and supporting recovery was heavy. The safety threat of rockburst impact and falling rocks to personnel and equipment has been basically controlled.

### Criteria of rock burst prevention and control in TBM construction

Common engineering practices show that even if there is good rock burst prevention and control technology, it is difficult to achieve good prevention and control effects if the technical scheme cannot be selected and implemented according to certain theoretical criteria. For example, the support cannot be completed at the right time, or the technical scheme cannot be selected according to the rock burst grade. Therefore, prevention and control of rockburst must first consider the characteristics of TBM construction and the law of rockburst occurrence and follow the correct concept and theoretical criteria of rockburst prevention and control. Four theoretical principles of rock burst prevention and control are proposed based on the characteristics of TBM construction, the characteristics and laws of rock burst occurrence and the practical experience of rock burst prevention and control in engineering.

1. Construction speed control criterion. The construction speed control as used here refers to controlling the footage every day or within a certain period of time. On the one hand, as mentioned above, the construction speed of a TBM has a significant impact on the location of strong rockbursts. On the other hand, considering the space–time effect of rockburst, if the construction speed of TBM is too fast, the supporting prevention and control measures are too late to be completed before rockburst occurs. Regardless of how good the prevention and control technology is, it will still have the consequences of rockburst and bring the risk of equipment and personnel injury. The large amount of slag removal restricts the footage speed. Therefore, according to the occurrence level of rockburst and the characteristics of support prevention and control technology, it is important to actively control the driving speed, and the construction speed control criteria should be followed.
2. Risk control criteria. The concept of risk as used here refers to the risk brought by rock bursts, that is, the safety risk of rock bursts to TBM construction personnel and equipment and the risk of the impact of TBM construction speed, construction period and trapping. The corresponding prevention and control technical measures shall be taken according to the magnitude of risk hazards and the probability of occurrence. This is different from the control concept of the fault fracture zone. It is often feasible and effective to implement technical measures such as support after the tail of the TBM shield is exposed in the fault fracture zone. For rockburst, whether it is the result of rockburst prediction theory or the result of rockburst monitoring, it gives a possibility of rockburst and a general level prediction. It ’cannot reach and achieve a very accurate forecast level. However, serious consequences will result if technical measures are not taken in time before the rock burst. Therefore, even if it is proven that there is no real rockburst in the tunnel section at this location after excavation, there is a high probability of rockburst as long as it is judged according to the prediction results and the experience of similar rockburst surrounding rock conditions in the early stage of this project. It is necessary to take corresponding support and other technical measures to prevent and control rockbursts. This step represents a risk control measure. It cannot be considered that such prevention and control measures are waste. Simultaneously, the corresponding construction period and cost loss must be considered and recognized. Therefore, the prevention and control of rockburst is a preventive measure taken in advance according to the possibility of occurrence, and it is necessary to follow the risk control criteria of rockburst.
3. Temporal and spatial control criterion. Engineering practices show that rockburst may occur instantly or in a short time after excavation, which is called instant rockburst. This type of rockburst mainly occurs in the tunnel face and TBM cutter head or shield area. Rockburst may also occur after a period of delay after excavation. As mentioned above, the probability of such a rockburst occurring within 24 h is very high. Individual occurrence over a longer period of time. The location is mainly concentrated in the TBM host area. For immediate rockburst, rockburst mostly occurs in the range of the tunnel face and shield body. We can best leverage the characteristics of the TBM host structure to implement corresponding prevention and control technical measures. It is also necessary to design the cutter head, cutter, bucket teeth and shield robustly. For a rockburst with a long lag time, it is necessary to fully grasp the temporal and spatial law of rockburst, coordinate the driving footage and support speed of the TBM, and actively control the footage speed to control the location of the rockburst. Support measures should be implemented in proper time and space before TBM rockburst, and necessary personnel and equipment protection design should be made for the TBM host area. Therefore, the prevention and control of rockburst should consider the space–time effect and follow the space–time control criterion.
4. Graded control criterion. Different surrounding rock conditions have different rock burst grades. Rockbursts are generally divided into no rockburst, slight rockburst, moderate rockburst, strong rockburst or even extremely strong rockburst. Different rockburst grades have different hazards. Different prevention and control technical measures have different prevention and control effects. The resulting influence on the construction footage speed and the construction cost are different. The technical measures for the prevention and control of minor and moderate rockbursts may not solve the fundamental problem of strong rockbursts and extremely strong rockbursts. Therefore, we cannot talk about the technical scheme of rock burst prevention and control in general. According to the rock burst grade, different rock burst prevention and control technical schemes should be selected. At present, despite some engineering experience in overcoming these issues, there remains a lack of systematic theoretical guidance on how to choose the technical scheme of rock burst prevention and control. It is thus necessary to establish a theoretical system for selecting prevention and control schemes at different levels. The rock burst graded control criterion should be followed in TBM excavation.

### Prevention and control technology of rock burst in TBM construction

A technical system for classified prevention and control of rock burst in TBM construction based on coordinated control of equipment excavation support is proposed here according to the influence law, space–time effect, available equipment structure and construction method characteristics of TBMs on rock burst, as well as the prevention and control objectives and guidelines of rock burst. Compared with open TBMs, shield-type TBMs generally more easily prevent and control rockbursts, and the control is relatively simple because the main engine area is protected by the shield and the back supporting part is supported by prefabricated segments. Therefore, the following mainly discusses the technical scheme of rock burst prevention and control with the open TBM.

#### Rockburst prevention and control technology of different grades

For slight rockburst, the conventional open TBM is equipped with an anchor drilling machine at the tail of the shield, which is convenient for anchor operation. Moreover, the combined support of the bolt and mesh can resist slight rockburst. The operation speed of the bolt and mesh support is also relatively fast. Basically, it can keep up with the TBM excavation; even if it cannot keep up with the excavation for a while it will generally not bring the safety threat of equipment and personnel and can even remedy the support. Therefore, it is not necessary to actively control the pure tunneling speed of the TBM, but it is necessary to ensure that the support follows tunneling. Rock burst prevention and control support may actually cause some delay to TBM construction speed, i.e., footage speed. If the rockburst occurs in front of the shield tail, it is a large-scale continuous rockburst. In this case, the use of anchor rods and steel mesh is invalid because it will cause a large amount of slag to be cleaned. The technical scheme of the combination of an arch frame and a steel bar row with a large spacing should be adopted. It not only ensures safety and reduces slag falling but also greatly improves the construction speed. According to the above analysis, the technical prevention and control scheme of minor rockburst is a combination of conventional TBM design and bolt mesh or large-spacing arch reinforcement row support and support following excavation without actively controlling the footage speed. For moderate rockburst, ordinary bolts and mesh support have difficulty resisting the impact of rockburst. The support by spraying concrete at the main engine of the TBM will pollute the main engine, and the strength will increase slowly. It is not a good choice unless concrete with low price, no rebound and high speed can be developed. Therefore, it is proposed that the continuous closed support method combining a steel arch frame and steel bar row is adopted for moderate rockburst. In this case, the TBM construction speed does not need to be actively controlled. Because the support may not keep up with the excavation, the construction speed will be passively controlled. In addition, to enable the TBM shield to store steel bars and achieve uninterrupted closed support, it needs to be designed as a shield with a storage interlayer. Thus, the medium rockburst prevention and control technical scheme is constructed, which includes the design of a storage bunker shield, the support of an arch reinforcement row with regular spacing and the passive control of excavation. For strong rockburst, past engineering practice shows that conventional steel arch support has difficulty resisting the impact of strong rockburst. Support is often damaged. In addition, a lot of slag cleaning work will be brought. It threatens the safety of equipment and personnel and seriously affects construction progress. Nevertheless, according to the analysis results of the characteristics and laws of rockburst, the active control of the daily footage in the case of strong rockburst has a significant impact on the location of rockburst. Therefore, most strong rockbursts can occur in the face and shield area by actively controlling the daily footage. In this way, not only the equipment and personnel behind the shield are protected. It is more important that the problem of rock burst protection with enormous impact energy can be transformed into the problem of landslides and broken zone support. Namely, the rockburst and its falling rocks fall in the area from the tail of the shield to the front face, and the cutter head and shield play a role in resisting the impact of rockburst. Small-spacing steel arches and steel bars can be combined at the tail of the shield to support rockburst and rockfall. On this basis, a strong rock burst prevention and control technical scheme is proposed, which combines the design of a robust cutter head shield, active control of footage speed and steel bar row support of a small spacing steel arch frame. Generally, the distance from the tunnel face to the shield tail is approximately 6 m, and more than 90% of strong rockbursts occur within 24 h. According to this law, the daily footage of TBM excavation can be actively controlled below 3 ~ 5 m. With the excavation process, the combined support of the steel arch frame and steel bar row at the tail of the shield follows closely. After the footage control index is completed, the machine can stop and wait. The spacing between steel arch frames is reduced to enable the crossing of TBM support shoes. If the smaller spacing cannot be crossed, concrete pouring can be used to smooth it. For extremely strong rockbursts, there are actually local extremely strong rockbursts in the strong rockburst tunnel section of the Han-Ji-Wei Water Diversion Project. The concept and technology of rock burst prevention and control can be controlled with reference to strong rock bursts. The specific footage speed index, arch spacing and model can be adjusted appropriately. Simultaneously, a water hammer drilling rig equipped with a TBM can be used to conduct advanced drilling to degrade the extremely strong rockburst energy. Then, the tunneling support method mentioned above is adopted for crossing. In addition, a double-type support TBM can also be selected. Applied with the traditional open TBM technique, this approach adds a steel sheet installer and an auxiliary propulsion system, which is closely behind the shield to support the steel sheet. This operation further improves the safety and construction speed of TBM crossing strong rockbursts. However, its disadvantage is its high cost.

**Figure 8:**
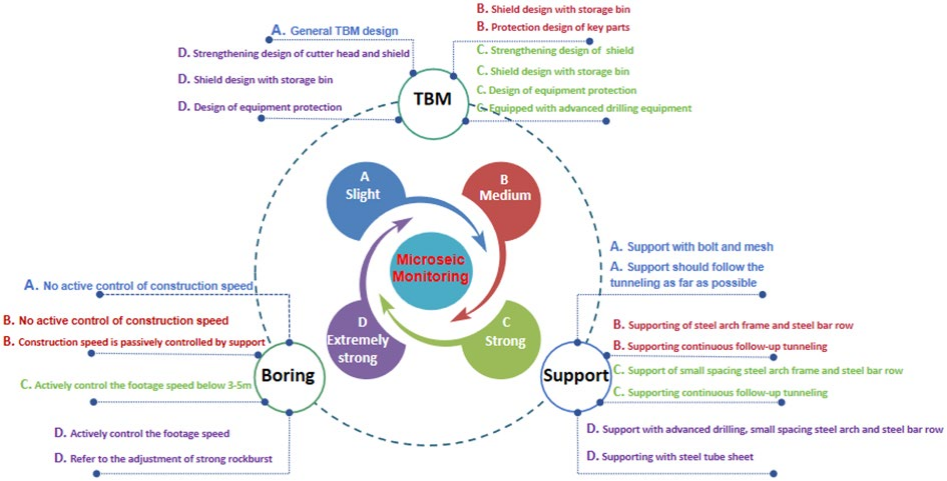
Different grades of rock burst prevention and control technical schemes.

The technical schemes of rock burst prevention and control with different grades are shown in Fig. 8.

#### Engineering application effect of graded rockburst prevention and control technology

The technical scheme of the prevention and control of minor rockbursts has been fully applied in the Xinjiang ABH project and Xinjiang EHe (EH) project. The ABH project in Xinjiang had 101 slight rockbursts by August 2019. The rock burst can be completely controlled by the support, and no equipment damage or casualties have occurred. Because of the compressive strength and integrity of the surrounding rock of the ABH project, it is easy to tunnel. The construction speed under slight rockburst is approximately 70–90% of that without rockburst. In the Xinjiang EH project, a large area of continuous minor rockburst to moderate rockburst occurred in front of the shield tail, and the slight rockburst adopted the technical scheme of combining long-distance arch frames and steel bars. In the field application of tunneling for several kilometres, the effect is clear, the safety is controlled, and the monthly footage can reach approximately 400 m.

In the ABH project and the YHJW project, the monitoring and prediction of rockburst microseisms include moderate rockburst, slight-moderate rockburst or moderate-strong rockburst, all of which adopt the abovementioned rockburst prevention and control technical scheme. From the start of TBM excavation in the ABH project to August 2019, there were 18 slight-moderate rockbursts and 32 moderate rockbursts. From excavation to November 2019, there were 84 slight-moderate rockbursts, 158 moderate rockbursts and 80 moderate-strong rockbursts in the YHJW project. By adopting the above prevention and control technical scheme, the support resisted the impact of rockburst, no casualties occurred, and the equipment was not greatly damaged. Moreover, this scheme achieved a relatively fast TBM construction speed, which is approximately 50%—70% of the construction speed that is achieved without rockburst. The daily footage is approximately 10 m when the steel arch frame and steel bar row are used for support in the ABH project.

In the Lingnan section of the YHJW project, with increasing burial depth, it began to enter the continuous strong rockburst area or even the extremely strong rockburst area after the TBM entered the second excavation section.

By the end of October 2020, the second excavation section had completed a 2075.3 m excavation, and there were 813 rock burst sections that restricted excavation and caused shutdown support, with a total length of 2023.6 m, accounting for 97.5% of the excavated length. The TBM successfully passed through the strong rockburst tunnel section of more than 2 km by adopting the prevention and control scheme of rockburst by actively controlling the daily footage. However, approximately 7% of the secondary delayed rockbursts occurred in the arch and bottom arch. There were 37 rock bursts with strong lag in the arch and 21 rock bursts with lag in the bottom arch, which caused damage to the supported structural system. However, delayed rockburst occurred in the supported area. The threat to personnel and equipment is greatly reduced, which mainly brings the workload of support recovery and slag removal. The average monthly footage of the TBM in a strong rock burst tunnel reaches 110 m, and the daily footage is approximately 3.5 m. The TBM safely passes through the long-distance strong rock burst tunnel section. Simultaneously, the TBM cutter head and shield also proved to be able to withstand the impact of strong rockburst. Since the TBM was originally designed according to the convention, the cutter head and shield were somewhat damaged, but not enough to cause fatal problems. The TBM cutter head and shield should therefore be designed robustly according to the prevention and control scheme of strong rockburst proposed in this paper.

The application of double-type support TBMs in the construction of plateau railways provides another technical means for TBMs to cross extremely strong rock bursts. There is no engineering case list of steel pipe segments in dealing with extremely strong rockburst, but the steel pipe segments in dealing with large deformation of soft rock in the Xinjiang EH project have been verified by engineering, and the effect is very good.

**Figure 9:**
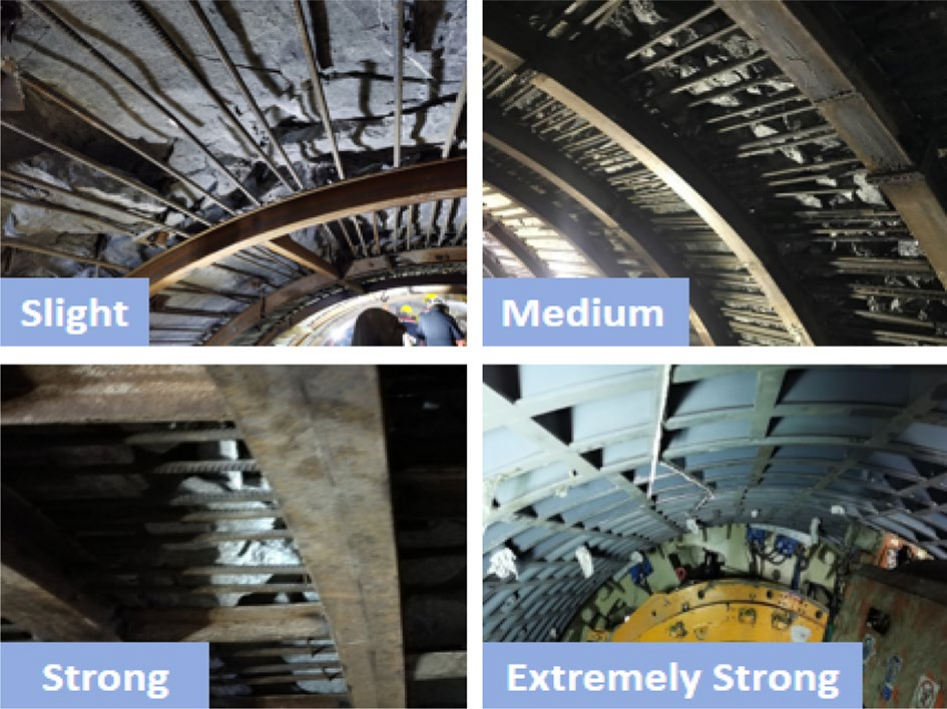
Engineering application effect.

The actual effect of engineering application of graded rockburst prevention and control technology is shown in Fig. 9.

## Discussion

In view of the above analysis results of rock burst characteristics and the proposed prevention and control technical scheme, the following aspects are discussed emphatically. (1) The projects with strong rockburst in the superdeep tunnel constructed by the TBM in China are mainly the Jinping II Hydropower Station Project, Shaanxi YHJW Project and Xinjiang ABH Project. The maximum burial depth of these three projects is more than 2000 m, and the tunnel section with burial depths above 1500 m accounts for a large proportion. The lithology of the Jinping II Hydropower Station is mainly marble, the ABH project is mainly siltstone and metamorphic mudstone, and the YHJW project is mainly granite, quartzite and diorite. They are representative to some extent. According to the literature^22–25^ about the Jinping II hydropower station project, the temporal and spatial characteristics of rockbursts are similar to those of the research results in this paper, showing a generally consistent law. However, this paper is based analysis of big datasets and obtains the temporal and spatial characteristics of different grades of rockburst more clearly according to the rockburst grades. The present study enriches the data on rock bursts in the TBM construction of superdeep tunnels. In this paper, the speed of footage is actively controlled; for example, the daily footage is 3–55 m. Most strong rockbursts can be controlled in the tunnel face and shield area. Results also show that the prevention and control strategy and technical scheme of strong rockburst according to the lag time of rockburst within 24 h and active control of daily footage proposed in this paper are well founded and reliable. (2) The prevention and control technology of rockbursts in this paper is not 100% controlled, and there are still a few rockbursts with long lag times. Strong support for these conclusions have been completed at this time. Even if a few supports are damaged to a certain extent, the safety of personnel and equipment has been greatly improved, and the amount of slag removal has been greatly reduced, thus greatly reducing the amount of labour. It could be common to think that according to the prevention and control strategy of actively controlling daily footage, the footage of TBM is too small, and the construction period is too delayed. In fact, in the TBM construction of the drainage tunnel of the Jinping II Hydropower Station, there was no explicit initiative to control the daily footage, and there was no shield with a storage bin to implement the reinforcement row support. The results showed that a large number of rockbursts occurred in the area behind the shield, strong rockbursts damaged the completed support, and a large number of rock blocks ejected by rockbursts needed to be cleaned. The workload of restoring support and cleaning slag was large, which greatly delayed the penetration speed of TBM construction. From July to November 2009, only 600 m was excavated, but the average monthly footage of the YHJW project reached 110 m. The bigger problem is that the rock burst prevention and control scheme of the Jinping II Hydropower Station makes the safety of equipment and personnel more threatened. (3) A series of rock burst prevention and control measures are found in both engineering practice and literature reports. For example, water spraying on the tunnel wall, anchor rod, mesh, shell-expanding prestressed grouting anchor rod, water-rising anchor rod, steel fibre reinforced concrete spraying, drilling to release stress and so on. These technical measures have a certain effect on the prevention and control of rockburst, but there are still many problems as a complete technical scheme for a TBM crossing a rockburst tunnel. These support methods may delay TBM excavation too much. Another more important aspect is that the support measures and shields are intermittent and difficult to implement quickly. The rockburst has happened before the support can be done. Therefore, it is necessary to study the supporting measures that can resist strong rockbursts and can be implemented quickly. Talking about support and other measures alone cannot prevent and control rockburst well. It is necessary to consider the characteristics of the TBM structure and construction method and, based on the understanding of the characteristics of rockburst, systematically conduct collaborative innovation and coordinated control from four aspects: monitoring, equipment, excavation and support to prevent and control rockburst to achieve better results. It is very important to note that in this paper, it seems that the strong rockburst still adopts the combination of a steel arch frame and steel bar row, but the arch frame is encrypted. However, the concept of rockburst prevention and control support has changed. Through design of the top shield containing the storage interlayer of steel bars and actively controlling the footage speed, most of the strong rockburst occurred from the shield to the tunnel face. Thus, the problem of supporting against rockburst impact is transformed into the problem of supporting broken rocks. The support avoids impacts caused by enormous energy, thus ensuring the integrity of the support. In addition, it should be noted that the support proposed in the above rockburst prevention and control technical scheme is a basic support method that can control rockbursts of different grades to ensure the safe and efficient crossing of TBMs. Other conventional supports, such as shotcrete in the back supporting position and consolidation grouting in the rockfall cavity at the back of the arch frame, can be implemented according to the requirements of engineering design, which will not be repeated here. In addition to the key support methods adopted in this paper, some new support technologies for preventing and controlling rockburst should be continuously studied, tested and applied, such as new buffer energy-absorbing arch supports, strong energy-absorbing protective nets, high-speed and high-toughness nonrebound concrete and other technical measures. The new stress release technology can also be explored. Simultaneously, it is necessary to solve that a few lagging rockburst supports are destroyed. In summary, it is still necessary to explore and innovate the prevention and control concept and technical scheme that can achieve satisfactory results in TBM construction speed and safety.

## Conclusions

This paper considers the YHJW project and Xinjiang ABH project as the main cases to study the characteristics and prevention technology of rock bursts in TBM construction of deeply buried tunnels. The results are compared with the Jinping II hydropower station project. This study covers all three TBM construction projects in China with a burial depth of more than 2000 m and strong rockbursts. The technical scheme of TBM construction prevention and control of classified rockburst is proposed through engineering practice exploration, data statistics and theoretical analysis. It is verified in engineering and draws the following main conclusions.

1. Different grades of rockburst have different effects on the construction speed, equipment and personnel safety of TBMs. In particular, strong rockbursts pose a great threat to safety. However, the safety risk of rock bursts in TBM construction can be controlled by adopting appropriate prevention and control concepts and technologies. By comparing and analysing the construction speed with and without rockburst, the construction speed of the TBM is reduced to approximately 70–90% of that without rockburst when the rockburst is slight. It decreases to approximately 50–70% in moderate rockburst and to approximately 25–50% in strong rockburst. Hence, the influence of rockburst should be fully considered in the surrounding rock classification, construction period and cost prediction of TBM construction.
2. The occurrence time of rockburst shows that approximately 20–40% of rockburst occurs within 10 h after excavation. The frequency of rockburst is the highest within 10–24 h after excavation. More than 90% of the rockbursts occurred within 24 h after excavation. Approximately 9% of strong rockbursts have a lag time of 24–48 h. From the location of the rockburst, more than 30% of moderate and strong rockbursts occurred in the area from the shield to the tunnel face. Above 90%, moderate and strong rockbursts occurred within 15 m behind the tunnel face. The scheme of actively controlling the penetration speed of the TBM can cause approximately 80–90% of strong rockbursts to occur in the area from the TBM shield to the heading face. This provides favourable conditions for TBM rockburst prevention and control. The penetration speed of TBMs actively controlled by strong rockbursts is generally approximately 3–5 m per day, which can be determined according to different engineering conditions.
3. On the basis of the influence of rockburst on TBM construction and its temporal and spatial characteristics. The structure, construction characteristics, safety and speed of the TBM are comprehensively considered. For four levels of rockburst, namely, slight, moderate, strong and extremely strong, this paper proposes the corresponding rockburst prevention and control objectives, rockburst prevention and control criteria (i.e., construction speed control, risk control, time and space control, hierarchical control) and rockburst prevention and control technical scheme. Therefore, a 4-4-4-4 theoretical and technical system of rock burst prevention and control based on monitoring, equipment, excavation and support is constructed.

In summary, using the prevention and control technical scheme proposed in this paper, one of the TBMs in the Xinjiang ABH project has safely and efficiently passed through all medium rock burst tunnels and completed the 14 km excavation of the bid section. The TBM in the Lingnan section of the YHJW project passes through the 18 km rockburst tunnel section. During the construction of the second tunneling section of the TBM, it passed through the 5 km continuous strong rockburst tunnel section, and the average monthly footage of the strong rock burst tunnel section reached 110 m. The proposed concept and technology of rock burst prevention and control have achieved good prevention and control effects in practical engineering applications, which can provide an important reference for subsequent ultradeep tunnel construction.

## Data Availability

The data used to support the findings of this study are available from the corresponding author upon request.
